# Outbreak of cutaneous anthrax associated with handling meat of dead cows in Southwestern Uganda, May 2018

**DOI:** 10.1186/s41182-022-00445-0

**Published:** 2022-08-06

**Authors:** Angella Musewa, Bernadette Basuta Mirembe, Fred Monje, Doreen Birungi, Carol Nanziri, Freda Loy Aceng, Steven N. Kabwama, Benon Kwesiga, Deo Birungi Ndumu, Luke Nyakarahuka, Joshua Buule, Caitlin M. Cossaboom, David Lowe, Cari B. Kolton, Chung K. Marston, Robyn A. Stoddard, Alex R. Hoffmaster, Alex Riolexus Ario, Bao-Ping Zhu

**Affiliations:** 1 Uganda Public Health Fellowship Program, Kampala, Uganda; 2 Ministry of Health, Kampala, Uganda; 3 National Animal Disease Diagnosis and Epidemiology Centre, Ministry of Agriculture, Animal Industry and Fisheries, Entebbe, Uganda; 4 National Centre for Emerging and Zoonotic Infectious Diseases, US Centers for Disease Control and Prevention, Atlanta, USA; 5 Uganda Virus Research Institute, Entebbe, Uganda; 6 Division of Global Health Protection, US Centers for Disease Control and Prevention, Atlanta, USA

**Keywords:** Anthrax, Cutaneous, Zoonoses, *Bacillus anthracis*, Outbreak, Uganda

## Abstract

**Background:**

Anthrax is a zoonotic infection caused by the bacteria *Bacillus anthracis*. Humans acquire cutaneous infection through contact with infected animals or animal products. On May 6, 2018, three cows suddenly died on a farm in Kiruhura District. Shortly afterwards, a sub-county chief in Kiruhura District received reports of humans with suspected cutaneous anthrax in the same district. The patients had reportedly participated in the butchery and consumption of meat from the dead cows. We investigated to determine the magnitude of the outbreak, identify exposures associated with illness, and suggest evidence-based control measures.

**Methods:**

We conducted a retrospective cohort study among persons whose households received any of the cow meat. We defined a suspected human cutaneous anthrax case as new skin lesions (e.g., papule, vesicle, or eschar) in a resident of Kiruhura District from 1 to 26 May 2018. A confirmed case was a suspected case with a lesion testing positive for *B. anthracis* by polymerase chain reaction (PCR). We identified cases through medical record review at Engari Health Centre and active case finding in the community.

**Results:**

Of the 95 persons in the cohort, 22 were case-patients (2 confirmed and 20 suspected, 0 fatal cases) and 73 were non-case household members. The epidemic curve indicated multiple point-source exposures starting on May 6, when the dead cows were butchered. Among households receiving cow meat, participating in slaughtering (RR = 5.3, 95% CI 3.2–8.3), skinning (RR = 4.7, 95% CI = 3.1–7.0), cleaning waste (RR = 4.5, 95% CI = 3.1–6.6), and carrying meat (RR = 3.9, 95% CI = 2.2–7.1) increased the risk of infection.

**Conclusions:**

This cutaneous anthrax outbreak was caused by handling infected animal carcasses. We suggested to the Ministry of Agriculture, Animal Industry and Fisheries to strengthen surveillance for possible veterinary anthrax and ensure that communities do not consume carcasses of livestock that died suddenly. We also suggested that the Ministry of Health equip health facilities with first-line antibiotics for community members during outbreaks.

## Introduction

Anthrax is a bacterial zoonotic infection caused by *Bacillus anthracis* (*B. anthracis*) and is transmitted to humans through contact with animals and animal products, such as meat, skins, and hide [1]. Approximately 20,000 to 100,000 cases of human anthrax are reported annually with most occurring in poor or rural areas which usually have low vaccination rates for livestock. Moreover, 64 million poor livestock farmers live in risk areas for anthrax. The disease is rare in developed countries due to the high vaccination rates in livestock [2].

Human anthrax infections may be cutaneous, inhalational, injection-associated, or gastrointestinal, based on exposure and routes of transmission. Cutaneous anthrax is the most common form with an incubation period of 1–7 days. This type of anthrax is the least dangerous, however, can become fatal if left untreated accounting for a case-fatality rate of up to 20% [3].

Uganda has reported anthrax outbreaks in the past among humans, livestock, and wildlife. These outbreaks have mainly been reported in areas. where people commonly keep livestock majorly within western, eastern and northern Uganda [4–6]. Vaccination rates of livestock in sub-Saharan Africa are very low, 0–6%, hence the risk of infection with anthrax in livestock is high [2]. More so, animal carcasses that die due to anthrax are often consumed as food or unsafely disposed of hence the risk of human infection and continuous exposure of spores to livestock is eminent [7].

On 6 May 2018, a farmer in Engari Sub-county, Kazo County, Kiruhura District, Uganda, reported the sudden death of three cows to the Engari Sub-county chief. Workers on the farms had reportedly opened the carcasses, butchered the meat, and sold it to traders in Engari and the neighboring Kanoni sub-county. On 12 May 2018, the Engari sub-county chief received alerts about persons who had developed symptoms consistent with cutaneous anthrax. The human patients had developed symptoms after various exposure to the dead cows. A team of field epidemiologists from the Uganda Public Health Fellowship Program, National Animal Disease Diagnosis and Epidemiology Centre, Ministry of Agriculture, Animal Industry and Fisheries, Ministry of Health, and Uganda Virus Research Institute traveled to Kiruhura District to investigate and determine the magnitude of the outbreak, identify exposures associated with illness, and recommend evidence-based control measures.

## Methods

### Study area

**Fig. 1:**
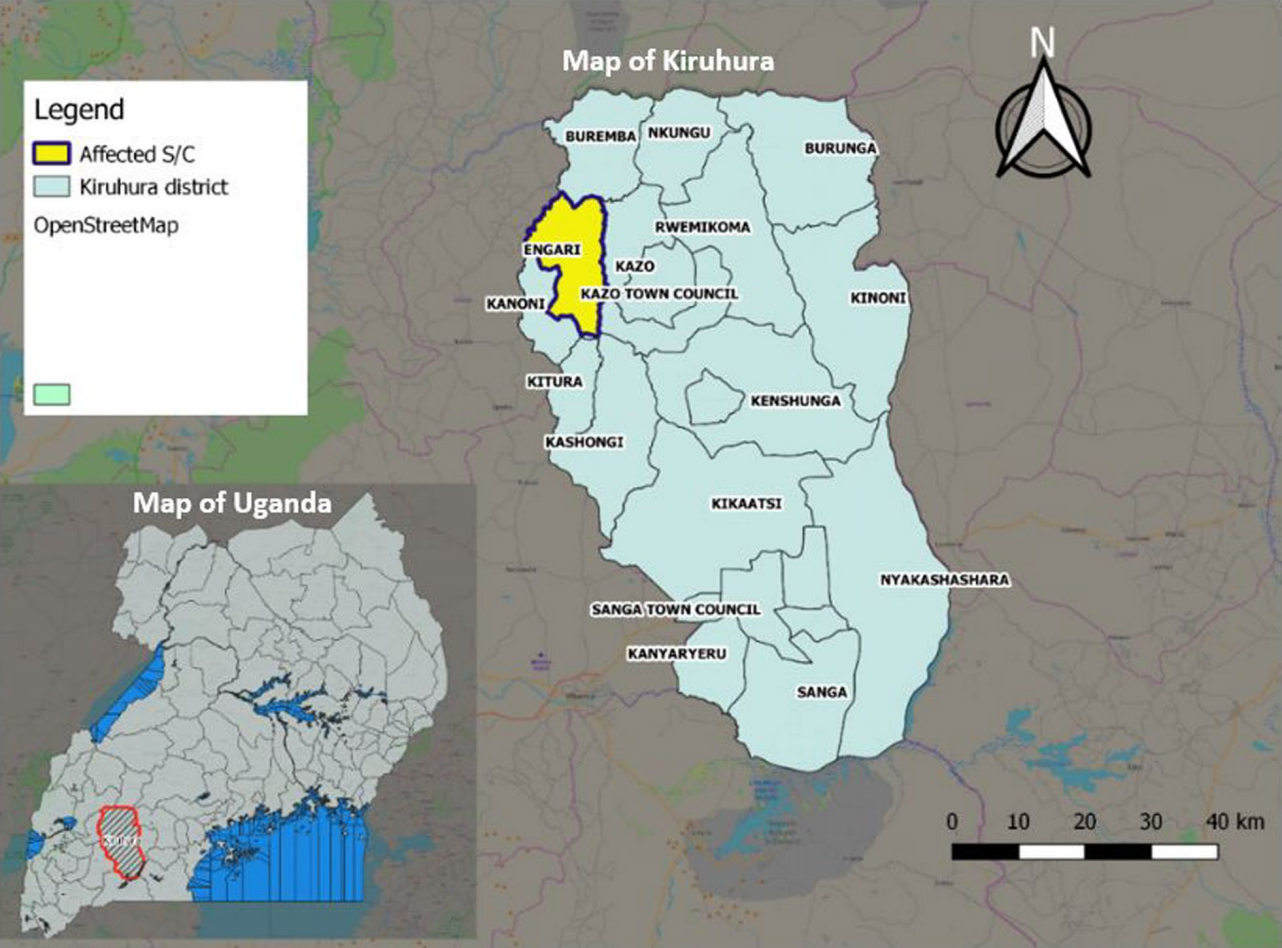
Showing the affected sub-county in Kiruhura District, May 2018

The investigation was conducted in Kiruhura District, located in the southwestern part of Uganda (Fig. 1; designed using Quantum Geographical Information System version 2.18.23). The district forms part of the cattle corridor area of Uganda which is a broad zone stretching from southwestern to northeastern Uganda, dominated by pastoral rangelands [8], and the major economic activities include livestock (cows, goats, and sheep) and farming.

### Case definition and case finding

We defined a suspected cutaneous anthrax case-patient as the presence of skin lesions (e.g., papule, vesicle, or eschar) in a person residing in Engari Sub-county, Kiruhura District from 1 to 26 May 2018. We defined a confirmed anthrax case-patient as a suspected case-patient with a clinical sample (skin lesion, vesicles, or blood) testing positive for *B. anthracis* by polymerase chain reaction (PCR). To identify cases, we visited and reviewed medical records at Engari Health Centre and private clinics in the affected sub-county. With the help of community health workers, we conducted active case-finding in the community and updated the line list. In addition, we collected information on demographic characteristics and symptom presentation.

### Descriptive epidemiologic analysis

We described case-patients by person, place, time, and clinical characteristics. Using population data obtained from the Uganda Bureau of Statistics, we computed attack rates by sex, age group, and village. We also described ill livestock by date of symptom onset, age, date of death, and village. Other farms also reported deaths of various livestock, including cows and a goat, although they had not been linked to any of the cases. We interviewed livestock owners about the symptoms of their dead livestock before death.

### Hypothesis generation

We interviewed 22 case-patients who were admitted to Engari Health Centre. We asked about various exposures to livestock, including cows and goats, during 1–26 May 2018 in Kiruhura District.

### Retrospective cohort study

We conducted a retrospective cohort study, because the possibility of exposure in a household was random, since all members except infants could have had contact with raw meat. For this reason, a cohort was formed from the members of all households receiving any of the meat from the dead cow. We used a structured questionnaire to gather demographic characteristics (age, sex, and occupation) and potential exposures. We defined the effective exposure period to be 1–12 days before the onset of symptoms (i.e., between the minimum and maximum incubation period for cutaneous anthrax).

### Laboratory investigations

We collected eyelid tissue and hide specimens from ten carcasses (nine cows and one goat) including the three implicated cows. To test animal specimens in the field, we used the InBios Active Anthrax Detect (AAD) Rapid Test (InBios, http://www.inbios.com), a lateral flow assay that detects the capsular polypeptide of *B. anthracis.* The AAD Rapid Test is a point-of-care test originally developed as a diagnostic aid for human inhalation anthrax. It has shown promising results for use as a field test for presumptive identification of *B. anthracis* in animal carcasses in the field and is available for investigational or research use only [9–12]. We obtained swab samples by swabbing the skin specimens collected in the field. We performed the AAD Rapid Test in the field on tissue and skin swab samples collected from the carcasses, following the standard protocol provided by InBios (S. Raychaudhuri, InBios, pers. communication, 2016 May 10). Briefly, tissue samples were suspended in 600 µL of sterile phosphate-buffered saline (PBS) and were vortexed for 10 s. After pipetting repeatedly, 10 µL was applied to the AAD Rapid Test cassette. For skin swabbed exudate samples, we transferred 10 µL of fluid to the cassette directly without PBS.

Confirmatory real-time PCR (qPCR) testing was performed on all field samples at the Uganda Virus Research Institute (Entebbe, Uganda) on a Stratagene MX3000P PCR machine following a standard protocol [12]. Specimens were subsequently shipped to the Centers for Disease Control and Prevention (Atlanta, Georgia, USA) for confirmatory testing using culture, qPCR, and immunohistochemistry. Tissue and skin swab samples were processed and inoculated into sheep blood agar or heart infusion broth, then incubated at 37 ºC for 24 h. DNA extractions were performed on specimens using the QIAGEN Blood Mini Kit (https://www.qiagen.com). The resulting DNA was tested using the Laboratory Response Network’s RT-PCR for *B. anthracis* as previously described [11]. We processed formalin-fixed tissue samples (ear and/or eyelid biopsies) from nine cows and one goat, embedded them in paraffin, and stained them with hematoxylin and eosin, Lillie–Twort Gram stain, and Warthin Starry silver stain. We performed immunohistochemistry assays using mouse monoclonal antibodies targeting the *B. anthracis* cell wall and capsule using an immunoalkaline phosphatase polymer system, as previously described [13, 14].

### Trace forward and environmental investigations

We visited households, where the implicated dead cows and goats were slaughtered to gather information on how the meat and other parts were distributed. We visited nine villages, where case-patients were reported and observed the appearance of pasture on which the livestock was grazing and whether there was any dead livestock. We also observed the different selling points for meat and asked about livestock movements and trading in the affected district.

### Data management and analysis

We managed the data in Microsoft Excel and performed the analysis in Epi Info 7.2. Basic analyses were done to obtain frequencies and proportions. We obtained population data for the administrative areas from the sub-county headquarters and used it to calculate attack rates. We computed risk ratios to establish the risk factors associated with anthrax infection.

## Results

**Fig. 2:**
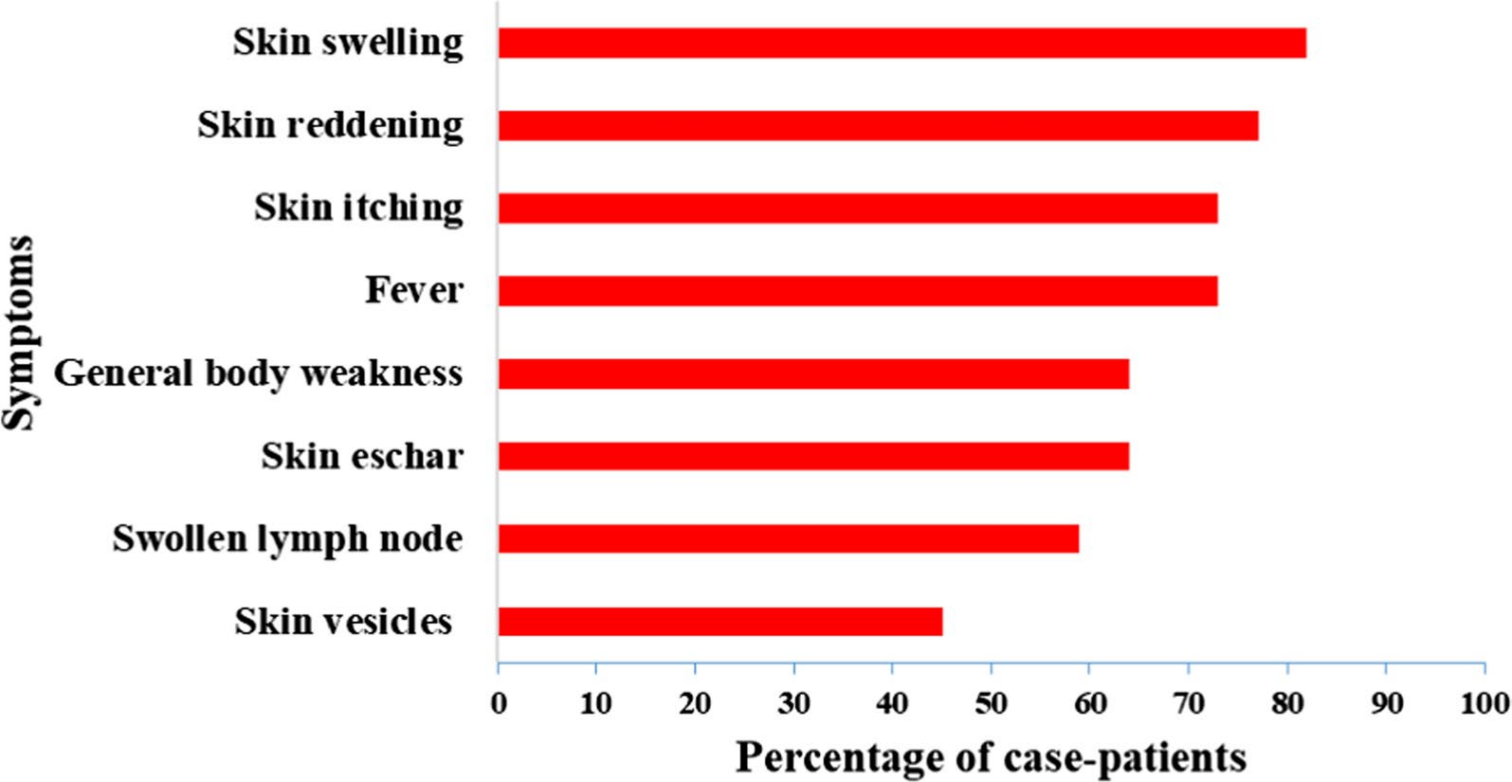
Symptoms presented by 22 case-patients during the anthrax outbreak in Kiruhura District, May 2018

We identified 22 cutaneous anthrax case-patients. No patients with signs or symptoms of gastrointestinal anthrax were reported. Twenty (91%) case-patients were males; there were no fatal cases. Case-patients came from 20 farms, all in the Engari Sub-county. Cases were mostly clustered in the northern part of Engari sub-county. Twenty (91%) case-patients’occupation were related to livestock. The mean age of case-patients was 27 years [Standard Deviation (SD) ± 14 (range 4–64)]. All patients had at least one skin manifestation; 68% had a skin eschar (Fig. 2). The overall attack rate in the affected villages was 3.9/1,000 (Table 1). Case-patients aged 20–39 years (AR = 7.8/1000) were most affected. Males were more affected (AR = 7.3/1000) than females (AR = 0.69/1000) (p < 0.0001). Among eight affected villages, the median attack rate was 3.5/1000 (range 1.2–9.2).

**Table 1:** Attack rates by sex, age, village among case-patients during the cutaneous anthrax outbreak in Kiruhura district, May 2018

Variable	Frequency (*N* = 22)	Percent (%)	Population	Attack rate/1000
Sex^a^				
Male	20	91	2758	7.3
Female	2	9	2913	0.69
Age^a^				
0–9	1	4.5	1815	0.55
10–19	5	23	1361	3.7
20–39	12	55	1531	7.8
40–59	3	14	680	4.4
60 +	1	4.5	283	3.5
Village				
Rupyani	10	45	1088	9.2
Kantaganya	3	14	620	4.8
Bukiro 2	2	9.1	422	4.7
Nyamuhirwa	1	4.5	227	4.4
Kashitamo	2	9.1	763	2.6
Kitongole	2	9.1	1091	1.8
Bukiro 1	1	4.5	634	1.6
Imiramiringa	1	4.5	826	1.2
Total	22	100	5671	3.9

^a^Sex- and age-specific populations were estimated based on age distribution in Engari subcounty

**Fig. 3:**
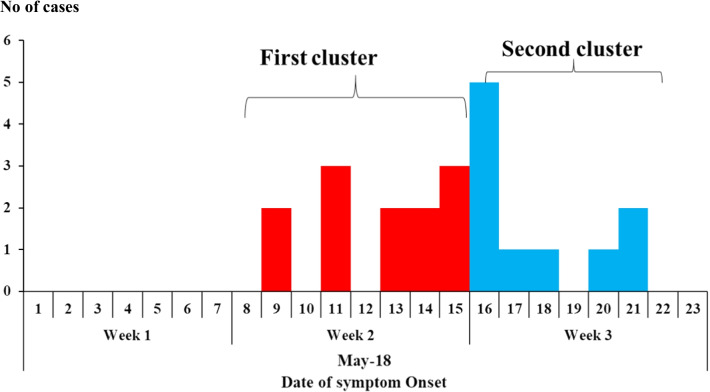
Overall epidemic curve showing the distribution of case-persons over time and points of exposure during the anthrax outbreak in Kiruhura District, May 2018

Case onsets occurred from 9 to 21 May 2018 (Fig. 3). All 22 (100%) case-patients reported exposure to dead livestock. Livestock carcasses prepared for meat sale in Uganda undergo several processes. These include removing the head and opening the carcass (‘slaughtering’), removing the skin of the carcass (‘skinning’), removing internal organs/waste, such as the offal (‘cleaning the carcass’), and cutting the carcass into pieces (‘cutting’). After the carcass is cut into pieces, butchers buy the meat pieces and carry them to their butcheries or meat stores, where they further cut and weigh meat into kilogram and half kilogram pieces for sale to the consumers (‘butchering for sale’). Beyond the reported livestock deaths, we also heard anecdotally that other livestock had died on different farms within the same sub-county.

Of the 22 case-patients, 14 (64%) participated in skinning and cleaning the carcasses of the three cows found dead on 6 May. Thirteen (59%) carried meat cut from those cows, and ten (45%) butchered the meat for sale. The overall epidemic curve shows two point-source outbreaks. Persons in the first cluster of cases participated in butchering the first two of the three cows on May 6, while persons in the second cluster of cases participated in butchering the meat of the third dead cow on 9 May (Fig. 3).

### Retrospective Cohort study findings

During our retrospective cohort study, we enrolled 95 persons. Slaughtering (RR = 5.3, 95%CI 3.2–8.3), skinning (RR = 4.7, 95% CI 3.1–7.0), cleaning the carcass (RR = 4.5, 95% CI 3.1–6.6), and carrying meat of the dead cows (RR = 3.9, 95% 2.2–7.1) increased risk of cutaneous anthrax (Table 2).

**Table 2:** Exposure factors associated with anthrax during the outbreak in Kiruhura District, May 2018

Risk factor	AR (exposed)	AR (unexposed)	Risk ratio	95% CI
Slaughtering dead cows	11/11 (100)	11/62 (17.7%)	5.6	3.2–8.3
Skinning	7/7 (100)	13/60 (21.7%)	4.6	3.1–7.0
Cleaning waste	7/7 (100)	13/60 (21.7%)	4.6	3.1–6.6
Carrying meat^a^	10/15 (67)	11/62 (17.7%)	3.8	2.2–7.1

^a^On shoulders, arms, on a stick, bicycle and motorcycle

### Environmental assessment findings

Members of the affected community reported that they had experienced a long dry season, lasting over 6 months (November 2017–April 2018) without rainfall [17]. In preparation for the rainfall, a central valley dam was constructed on 28 February–2 March 2018 in Rupyani village for water harvesting; both cows and goats reportedly drank water from the reservoir provided by the dam. The dam was dug by a 32-year-old herdsman who developed wounds consistent with cutaneous anthrax on 10 March 2018 and died on 15 March. According to farm owners in the affected community, the deceased had dug water dams on multiple farms but did not use standard safety measures, such as disinfecting his gumboots, before visiting the next farm.

Conversely, livestock on the farms had grazed on dry pasture due to the dry season. According to the sub-county security officer, 45 animal deaths had been reported from 20 farms from 6 to 25 May 2018 which had experienced loss of vegetation due to the drought. Shortly after the dry season, heavy rain (10 mm) fell from 4 to 7 April 2018 [15, 16]. On 6 May 2018, three cows died of suspected anthrax in Rupyani village, 28 days following the rains.

Community members reported that meat from the cows was sold at less than one dollar per kilogram, which is less than half the price at which cow meat is normally sold. Approximately 800 exposed persons, including those involved in the processing of the dead cows and their products. The people who ate meat from the implicated cows received post-exposure prophylaxis twice per day (ciprofloxacin; 250 mg for children and 500 mg for adults taken over a month during 8–30 May 2018). This could have controlled the outbreak and reduced the number of cases of anthrax.

### Laboratory findings

We collected samples from ten livestock carcasses, including one goat and nine cows, from four affected farms in Kiruhura District. Eight samples were confirmed positive by culture, RT-PCR, and/or IHC positive. All the eight positive samples initially tested positive for anthrax by AAD Rapid Test (Table 3).

**Table 3:** Summary of anthrax diagnostic testing result, by carcass sampled, in Kiruhura District, Uganda, May 2018

Species	AAD rapid test	Culture	RT-PCR	Immunohistochemistry	
				Cell wall	Capsule
Bovine	+	+	+	+	+
Bovine	+	−	+	+	+
Bovine	+	−	+	+	+
Bovine	+	+	+	−	+^a^
Bovine	+	−	+	+	+
Bovine	+	−	−	+	+
Bovine	+	−	+	−	+^a^
Bovine	+	−	+	NT	NT
Caprine	−	−	−	−	+^a^
Bovine	NT	−	−	−	−

AAD Rapid Test, InBios Active Anthrax Detect Rapid Test (InBios, http://www.inbios.com)

*NT* not tested

^a^Immunoreactive for capsule, not cell wall, which is suggestive of *B. anthracis,* but not confirmatory

## Discussion

We investigated a cutaneous anthrax outbreak associated with handling dead cows and their products. Persons involved in processing cows that had died had an increased risk of cutaneous anthrax. Several clinical samples from humans and livestock tested positive for *B. anthracis*. Despite the meat from these animals being used for consumption, no apparent cases of gastrointestinal anthrax occurred.

The major cause of anthrax in humans is direct or indirect exposure to infected animal products, whereas the risk factors of anthrax among the animal population are host susceptibility, droughts followed by heavy rains and low levels of pastures hence animals graze close to the ground [1]. These factors were present most likely influencing the anthrax outbreak in both animals and humans. Previous investigations of outbreaks in Uganda that have been done recently also found the association of anthrax to handling of meat from animals that died suddenly [4, 6, 17].

During this outbreak, case-patients included farmers, butchers, and herdsmen. All were known to have had contact with livestock fourdays before symptom onset. Contact with livestock included skinning, slaughtering, carrying meat and cleaning the carcasses of the animals. These are mainly male-dominated roles which explains why males are usually the most affected sub-population during anthrax outbreaks [4, 6, 18].

There were no fatalities during this anthrax outbreak. Most case-patients were receiving treatment at the time of the investigation and other exposed persons were given post-exposure prophylaxis. This prompt response by the district task force explains why there were no fatalities. Cutaneous anthrax is usually fatal if not treated (3).

The main source of exposure to humans was handling carcasses of animals that had died suddenly. Despite the Uganda Disease Act that indicates that no animal slaughter/ meat sales should be done for any sick animal or during an outbreak, this is greatly violated in most communities in the country [19]. However, in the western part of the country, where the outbreak was reported, 95% of families earn their livelihood from livestock. As a result, farmers may try to recoup potential losses by selling meat from dead animals, even if they are aware of potential risks. Alternate approaches to discourage this practice, such as compensation for animals lost to anthrax, may be needed to successfully avoid future outbreaks.

There were community reports that meat from the implicated cows was sold more cheaply than normal meat. Despite the consumption of this meat, no gastrointestinal anthrax cases were reported. It is possible that cases did occur but were not reported or diagnosed due to the non-specific signs and symptoms of gastrointestinal anthrax [3, 20, 21]. Another possible explanation for the lack of gastrointestinal cases is the rapid deployment of post-exposure prophylaxis that may have successfully prevented people from developing symptoms of anthrax following exposure to infected carcasses [21]. In addition, implicated meat was smoked over an open fire for about 60 min and boiled for 90 min. This could have prevented infection within the gastrointestinal tract as observed during the outbreak investigation in Kween District [4]. All factors considered, exposure primarily occurred during the handling of the carcass and meat from animals that had died suddenly.

## Limitations

Surveillance of anthrax in humans and animals is challenging due to a lack of awareness and identification of cases. Some individuals may have experienced mild, non-specific signs and symptoms of anthrax but missed during case-finding. This may have contributed to an underestimation of the scope of the outbreak.

## Conclusions and recommendations

We report a cutaneous anthrax outbreak associated with the handling of livestock carcasses confirmed to be infected with *B. anthracis*. We suggested vaccination of all animals in the affected sub-county and the surrounding areas as well as safe disposal of dead animals. In addition, we suggested that the Ministry of Health and Ministry of Agriculture, Animal Industry, and Fisheries investigate potential anthrax hotspots throughout Uganda, vaccinate animals in areas, where the disease is endemic, and educate the public on the risks of eating meat from animals that died of an unknown cause. The use of a One Health approach with multi-sectoral stakeholder collaboration can facilitate this process.

### Public health actions

During the investigation, we provided health education in the affected communities to highlight the risks of consuming meat from animals found dead of unknown causes, collected specimens from suspect human and animal anthrax cases for confirmatory testing, and provided post-exposure prophylaxis to all persons who consumed meat from the dead cows. In addition, we trained farmers in the affceted sub-county on the safe disposal of dead animals.

## Data Availability

The study data belongs to the Uganda Public Health Fellowship Program. However, it is available from the corresponding author upon reasonable request and with permission from the Uganda Public Health Fellowship Program.

## Abbreviations

AR: Attack rate
CDC: Centers for Disease Control
LFA: Lateral flow assay
NADDEC: National Animal Disease Diagnostic and Epidemiology Centre
PCR: Polymerase chain reaction
PHFP: Public Health Fellowship Program
OR: Odds ratio
UVRI: Uganda Virus Research Institute
